# Supplementary figures and images for: Loss of cell–cell adhesion triggers cell migration through Rac1-dependent ROS generation
Source: Life Sci Alliance. 2022 Nov 29;6(2):e202201529. doi: 10.26508/lsa.202201529 (PMC9711860; doi:10.26508/lsa.202201529)

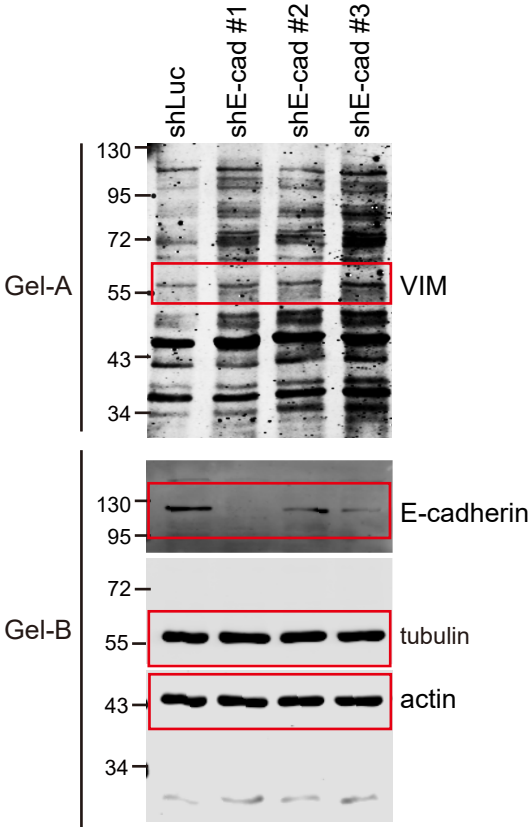

Figure 3A

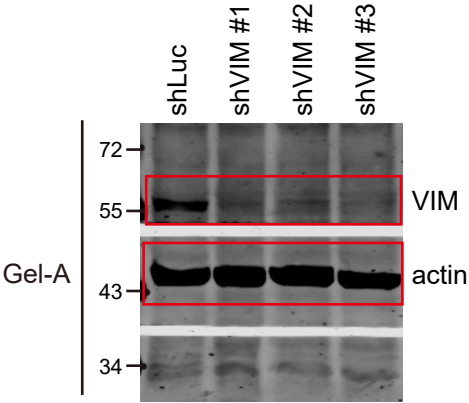

Figure 4D

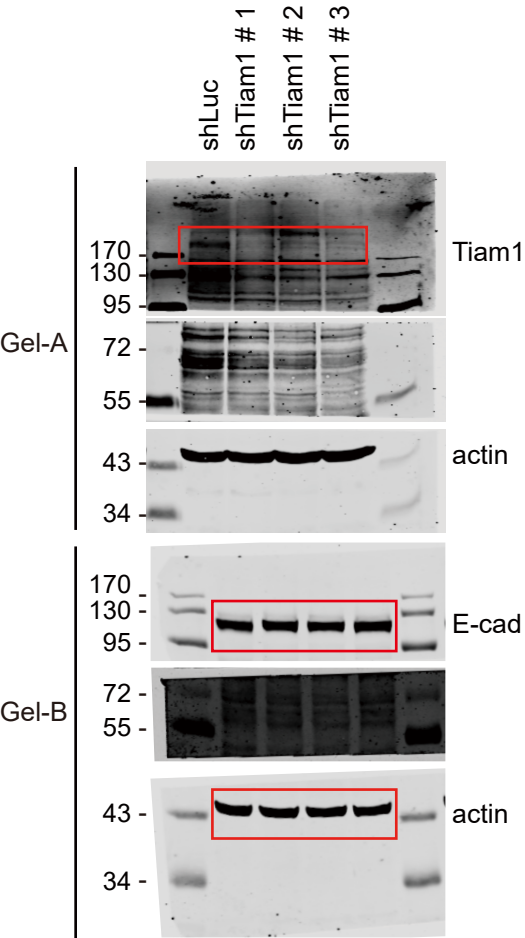

Figure 4H

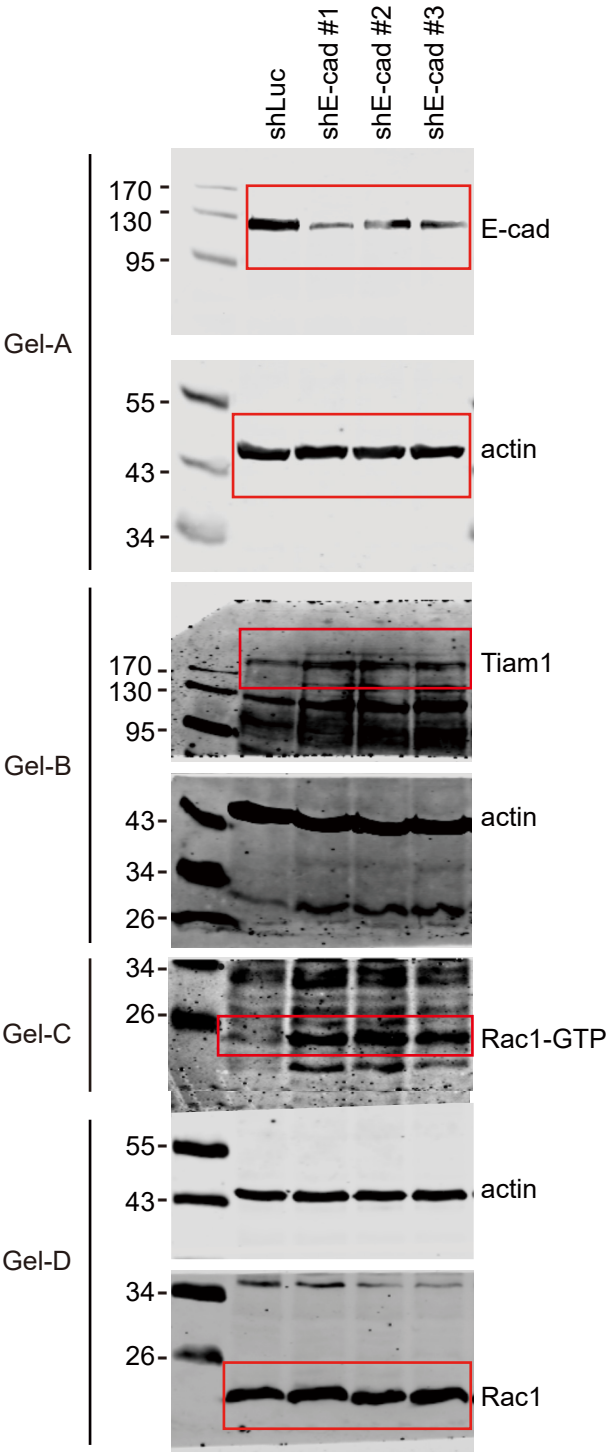

Figure 4J

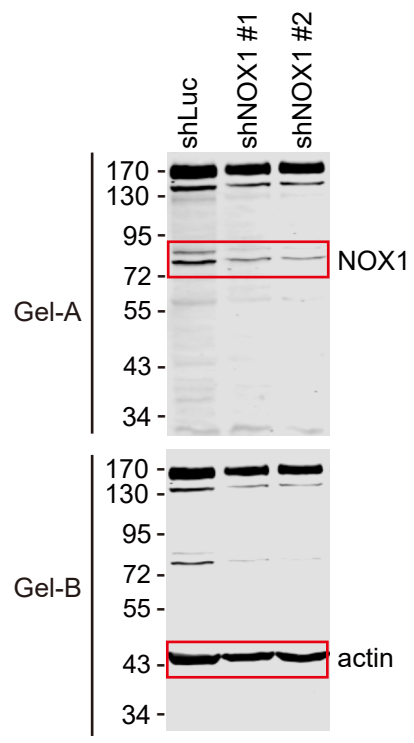

Figure 5A

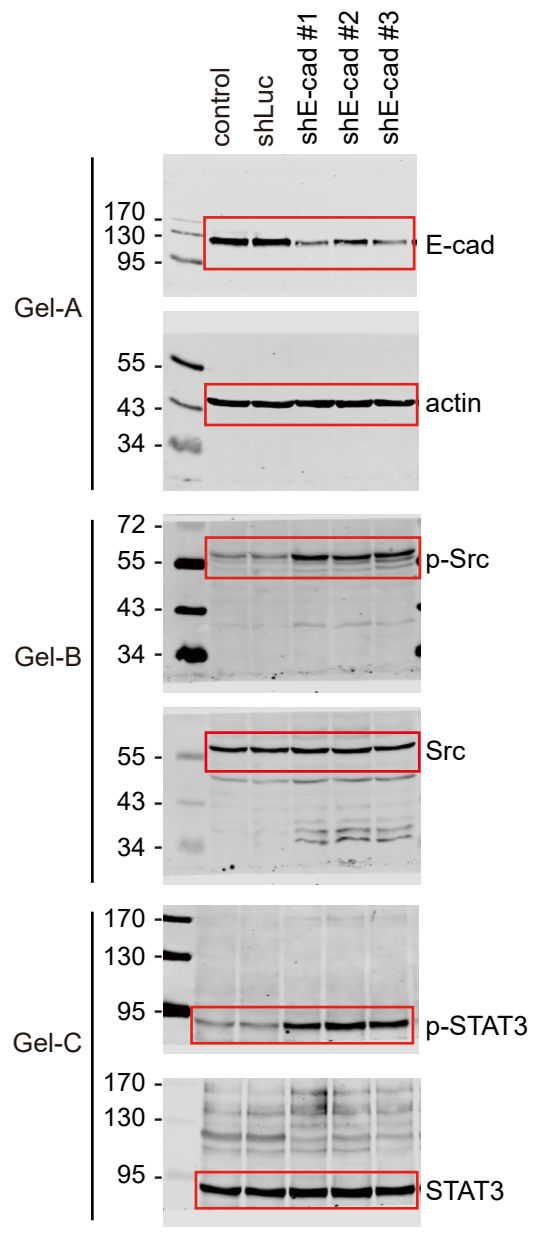

Figure 5B

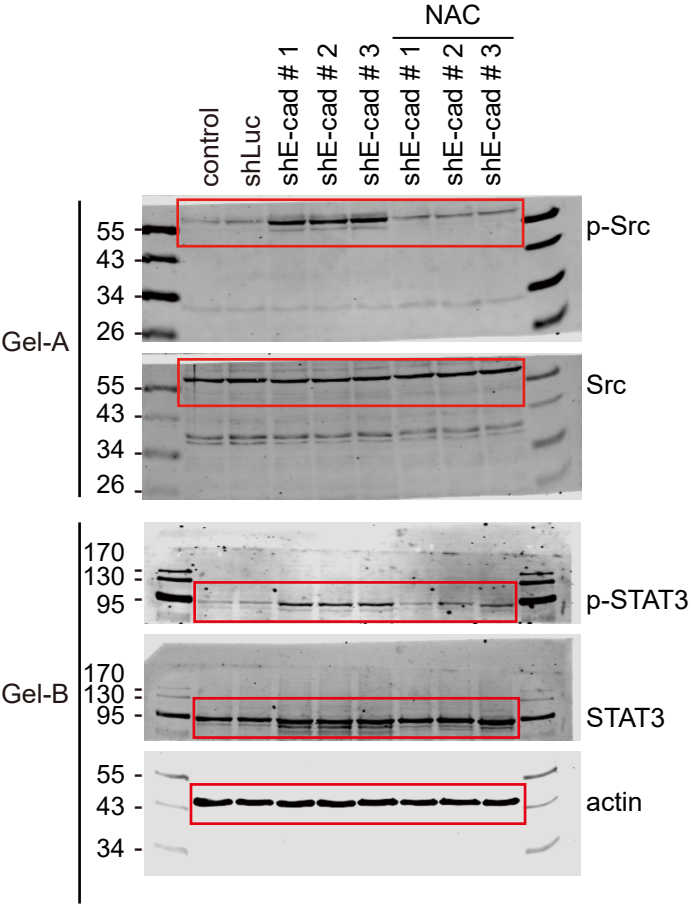

Figure 5C

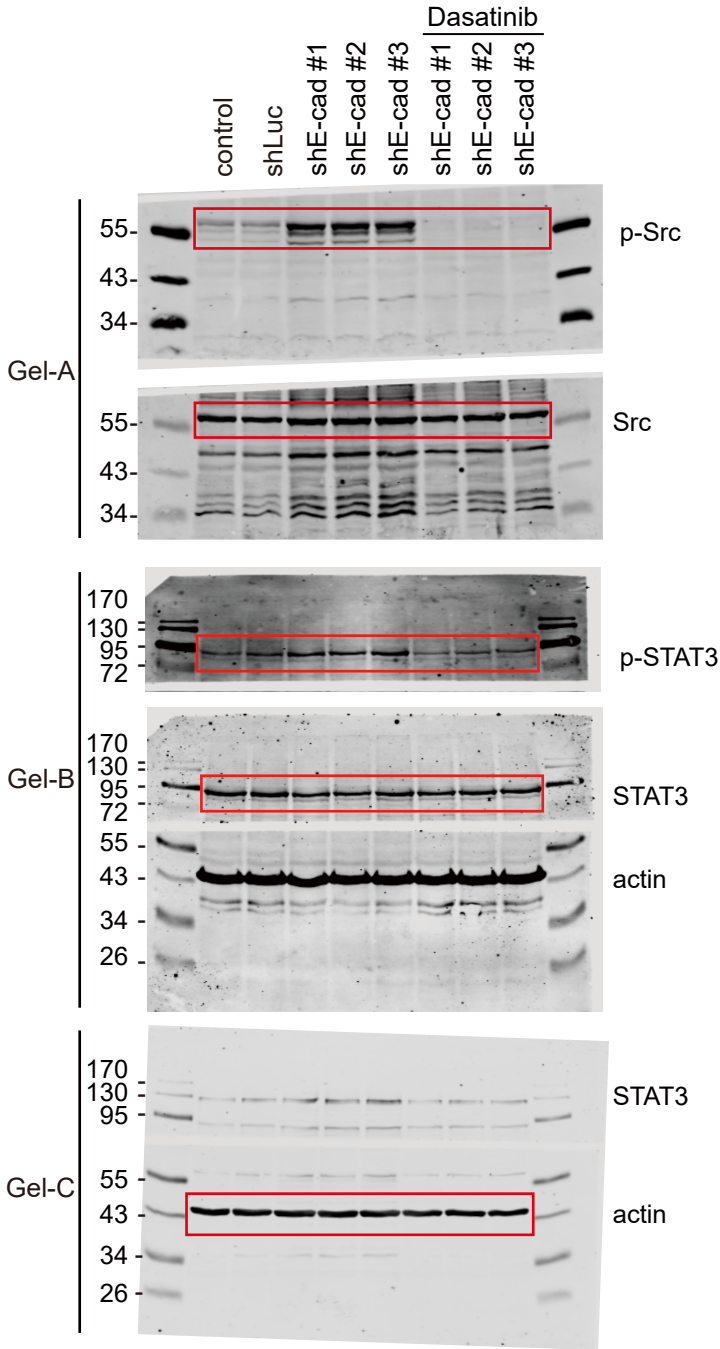

Figure 5D

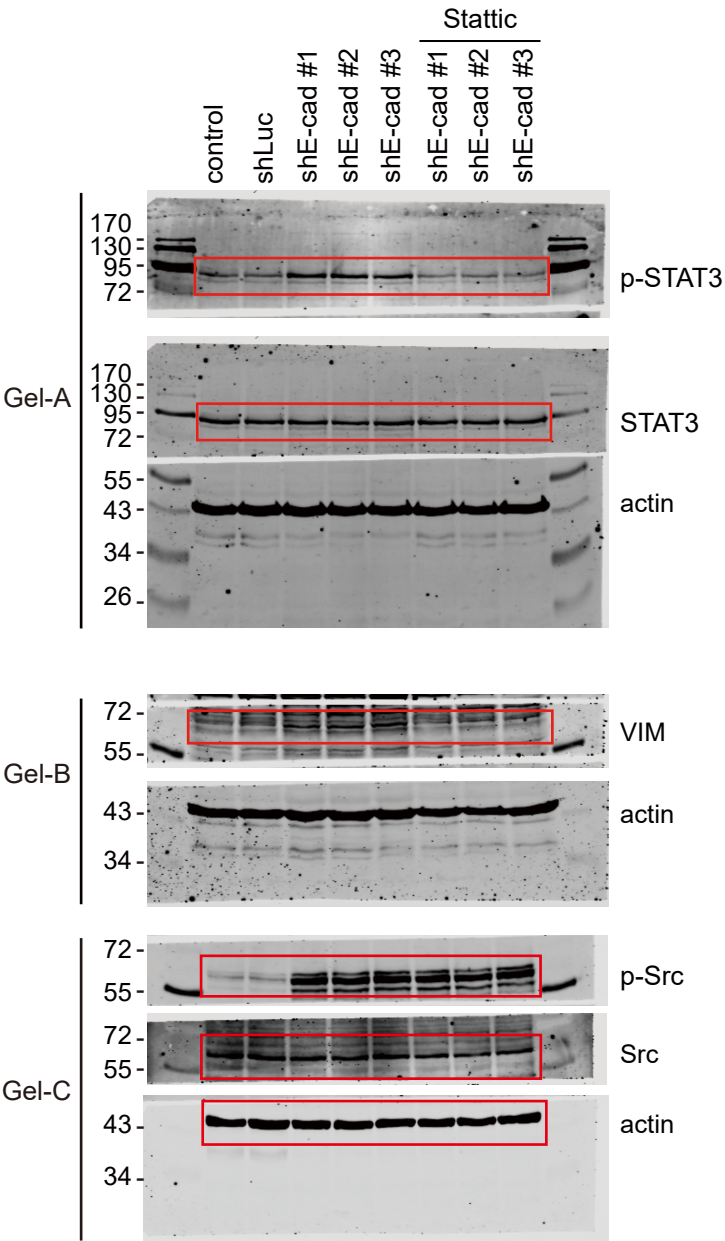

Figure S7B

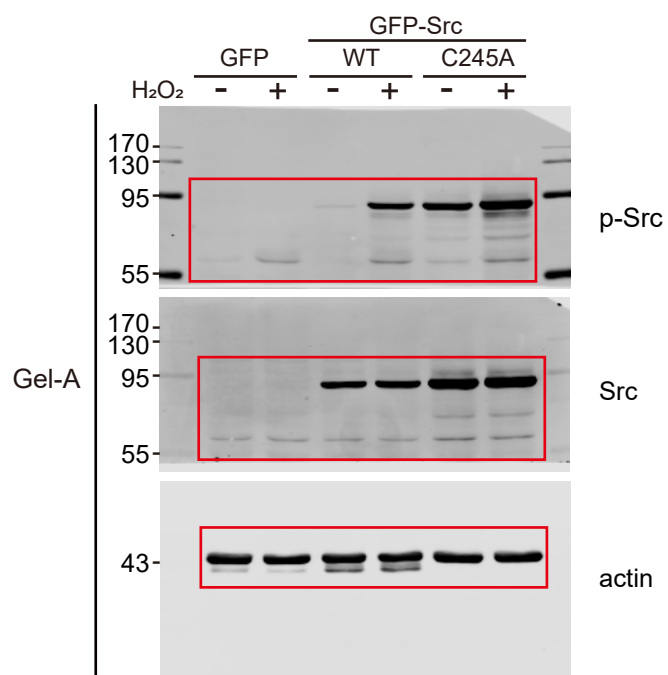

Supplement: Supplementary file 1 [file LSA-2022-01529_SdataF1_F3_F4_F5_FS7.pdf]
